# An Unusual Cause of Anemia and Encephalopathy

**DOI:** 10.4084/MJHID.2015.036

**Published:** 2015-05-01

**Authors:** Sanjeev Kumar Sharma, Dharma Choudhary, Anil Handoo, Gaurav Dhamija, Gaurav Kharya, Vipin Khandelwal, Mayank Dhamija, Sweta Kothari

**Affiliations:** Department of Hemato-Oncology and Bone Marrow Transplantation, BLK Superspeciality Hospital, New Delhi, India.

Although severe anemia can theoretically result in anemic hypoxia and can then lead to hypoxic encephalopathy, it is not a primary cause of encephalopathy. More frequently anemia can contribute with other multiple causes of encephalopathy such as infection, metabolic abnormalities, trauma, hepatic dysfunction, hypertension, toxins, etc. We present here an interesting case of recent onset anemia that was associated with an encephalopathy of unusual cause.

## Case

### Clinical history

A 49-years-old non-hypertensive, non-diabetic male was admitted to the hospital with the complaints of progressive weakness lasting three months. For the last two weeks, the patient also had drowsiness. The patient was hospitalized in the neurology ward for evaluation of encephalopathy. The patient became increasingly confused during his hospital stay. There was no history of fever, bleeding from any site, jaundice, head trauma or drug abuse. He was a non-vegetarian, non-alcoholic and non-smoker. Patient had a history of deep vein thrombosis one year back and was treated with warfarin.

### Clinical examination

On examination he had pallor; there was no icterus, cyanosis, pedal edema and lymphadenopathy. On per abdominal examination, there was no abdominal distension and no dilated veins over the abdomen. He did not have hepatosplenomegaly or ascites, and bowel sounds were normal. Central nervous system evaluation revealed him in altered sensorium with irritability and restlessness. There was no neck rigidity, and bilateral planters were flexor. Bilateral pupils were normal in size and well reacting to light. Examination of his respiratory and cardiovascular systems did not reveal any abnormality.

### Initial laboratory investigations

His hemogram showed pancytopenia with hemoglobin 7.6 g/dl, total leukocyte count 3.1×10^9^/l and platelet count 108×10^9^/l with normal differential count on peripheral smear. Mean corpuscular volume was 85 fl. His liver and kidney function tests were normal except for total serum protein 5.79 g/dl and albumin of 2.6 g/dl with reversal of albumin globulin ratio. His serum electrolytes including calcium and magnesium were in normal range. The workup for altered sensorium including cerebrospinal fluid examination and magnetic resonance imaging (MRI) of the brain was inconclusive. The screening for viral infections including HIV/Hepatitis-B virus/Hepatitis-C virus was negative. Blood culture was sterile. Peripheral smear did not show malarial parasites or schistocytes. His antinuclear antibody (ANA) and serum vitamin B12 and folate levels were also within the standard range. The thyroid stimulating hormone (TSH) was within normal range.

### Differential diagnosis

1. Infective causes of altered consciousness were evaluated and ruled out. Moreover, the patient did not have a fever and cerebrospinal fluid, and MRI findings were inconclusive. Also, the screening for infection including bacterial, malarial and viral pathogens was negative.
2. There was no vitamin deficiency/drug abuse/toxin or heavy metal exposure/head trauma.
3. Since the patient had a history of deep vein thrombosis, he was evaluated for paroxysmal nocturnal hemoglobinuria.
4. Bone marrow examination was planned considering myelodysplastic syndrome as a possibility.

### Further workup

**Figure 1:**
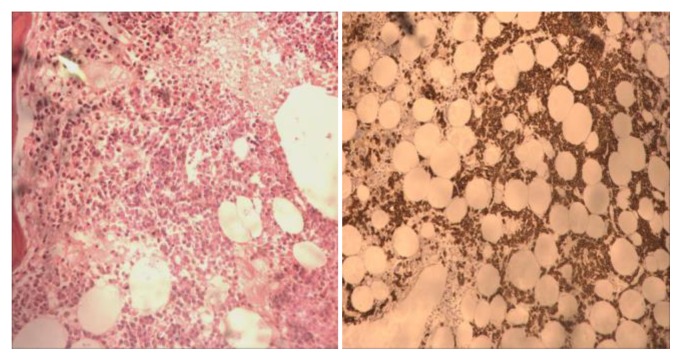
Bone marrow biopsy (200X) showing plasma cells in sheets and clusters (picture on the left side). CD 138 immunostain on bone marrow biopsy (100X) highlighting the plasma cells that are seen in sheets and clusters (picture on the right side).

Study for paroxysmal nocturnal hemoglobinuria (PNH) did not reveal any PNH clone. His bone marrow done for evaluation of cytopenias revealed sheets and clusters of plasma cells suggestive of multiple myeloma (Figure 1). Serum protein electrophoresis showed M band of 0.4 g/dl. Serum immunofixation electrophoresis showed kappa light chain only. Serum free light assay showed kappa-lambda ratio of 165. CT pulmonary angiography, performed for breathlessness and history of deep vein thrombosis, showed no evidence of pulmonary embolism, but the ribs showed multiple lytic lesions. A diagnosis of multiple myeloma was made. Liver-function tests and abdominal CT showed no evidence of hepatic dysfunction. Because of increasing altered sensorium and restlessness the patient required sedation and prophylactic intubation. Electrolytes, blood urea nitrogen, creatinine and calcium levels were unremarkable. Though the patient had multiple myeloma, the cause of altered sensorium could not be found, as all the usual causes of encephalopathy had been ruled out.

Further evaluation of encephalopathy showed that serum ammonia levels were high, 170 μg/dl (normal range 25–95 μg/dl).

### Treatment

He was started on anti-myeloma therapy with cyclophosphamide, bortezomib, and dexamethasone. Bortezomib was given 1.3 mg/m^2^ on day 1, 4, 8 and 11, dexamethasone 40 mg/m^2^ on day 1–4 and cyclophosphamide 500 mg once weekly. On fourth day patient’s sensorium improved, and he was extubated. His repeat ammonia levels done on day 4 of chemotherapy were 84 μg/dl and decreased to 50.6 μg/dl after three weeks of therapy. His karyotype showed tetraploidy. With continued treatment, the patient showed complete improvement in his sensorium and was discharged on day 22. His hemoglobin was 11.4 g/dl, total leucocyte count 4.7×10^9^/l and platelets 224×10^9^/l at the time of discharge. On day 60 of anti-myeloma therapy patient had no M-band in serum protein electrophoresis and kappa-lambda ratio was 2.14.

## Discussion

Central nervous system can be involved in various ways in multiple myeloma.^1^,^2^ Encephalopathy occurring in multiple myeloma is frequently due to metabolic disturbances related to the underlying plasma cell disorder, such as hypercalcemia and uremia.^1^ In addition, a direct invasion of CNS and leptomenings by the myeloma cells has also been reported.^2^ In our case hypercalcemia, and renal insufficiency were excluded by biochemical tests and MRI/CSF examination excluded CNS involvement by myeloma. Therefore, we considered hyperammoniemia a possible cause of encephalopathy. Hyperammonemia is involved mostly in the pathogenesis of hepatic encephalopathy that may present with an identical clinical syndrome, characterized by altered sensorium and impaired counsciousness.^3^ However, there were hepatic causes of hyperammonemia in our patient. Although it is not always possible to establish a right correlation between ammonemia level and neurologic symptoms, severe acute hyperammonemia causes a rapidly progressive, often fatal, encephalopathy with brain edema. Chronic milder hyperammonemia causes a neuropsychiatric illness.^3^ Therefore, prompt recognition can be lifesaving.^3^ Among the possible extra-hepatic causes of hyperammonemia, the literature also reports adult-onset inborn error of metabolism (a urea cycle disorder), carbamazepine or valproate use, and urinary tract infection with a urea-hydrolysing organism. Though rare, the underlying cause may be reversible, and potentially curable with the appropriate therapy of underlying disease.^3^,^4^

**Figure 2:**
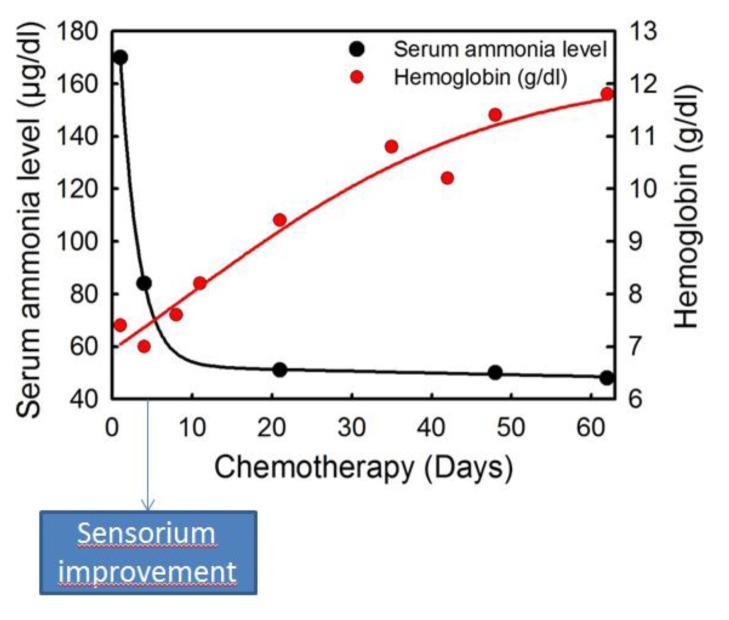
Serum ammonia level (μg/dl) showing dramatic reduction after starting chemotherapy. Black dots represent time points of measuring serum ammonia levels. Redline and dots represent the Haemoglobin level

Encephalopathy associated with hyperammonemia is a rare event in myeloma.^5^–^11^ However, it is a life threatening condition with an overall mortality of 40–48%.^5^,^10^,^11^ The diagnosis needs a high level of suspicion, and treatment should start as soon as possible. The cause of hyperammonemia in myeloma is not known though excess of ammonia production has been found in human myeloma cell lines,^12^ but also concomitant features could be significant. Chemotherapy directed against multiple myeloma is the most efficient treatment to achieve an improvement of neurological conditions in these patients. Thus, hyperammonemia should be considered in any patient with multiple myeloma and a low level of consciousness. Our patient presented with anemia and disturbance of consciousness. He was diagnosed to have multiple myeloma with hyperammonemia, and there was a dramatic and fast improvement in sensorium on treating him with chemotherapy. The improvement was clearly associated with the rapid decline in serum ammonia levels and progressive increase in hemoglobin level (Figure 2). The reduction in M band and free light chain ratio as well the rise of hemoglobin were late and then could not have any correlation with neurological improvement.

## Conclusion

The more frequent causes of altered sensorium in multiple myeloma include infections, hypercalcemia, uremia or dyselectrolemia, but when none of those could be found then hyperammonemia should be suspected, and treatment for myeloma should be started immediately. Therefore ammonemia should be tested in any patient with myeloma having an altered sensorium. The presentation of this case was unusual and misleading because the patient had pancytopenia and altered sensorium, without any routine biochemical data and radiological imaging suggesting any causes of encephalopathy. Only the bone marrow infiltration of plasma cells and the presence of osteolysis, while making evident the diagnosis of myeloma, suggested checking ammonemia. Anti-myeloma therapy was able to retrieve cytopenias and, by reducing ammonemia, also the altered sensorium.
